# Nutrition and maternal health: a mapping of Australian dietetic services

**DOI:** 10.1186/s12913-020-05528-4

**Published:** 2020-07-16

**Authors:** Shelley Ann Wilkinson, Elin Donaldson, Jane Willcox

**Affiliations:** 1 Department of Dietetics & Foodservices, Mater Health Services, Level 3, Salmon Building, Raymond Terrace, South Brisbane, QLD 4101 Australia; 2 Mater Research Institute, University of Queensland, South Brisbane, QLD 4101 Australia; 3 Dietetics and Human Nutrition, School of Allied Health, Human Services and Sport, La Trobe University, Melbourne, 3083 Australia

**Keywords:** Antenatal, Postnatal, Maternal health, Health services, Dietetics, Nutrition

## Abstract

**Background:**

Strong associations between diet and maternal and child outcomes emphasise the importance of evidence-based care for women across preconception, antenatal and postnatal periods. A 2008 survey of Australian maternal health dietetic services documented critically low resourcing with considerable variation in staffing levels and models of care. This study repeated the survey to examine resourcing in Australian maternal health services.

**Methods:**

A cross-sectional online survey was emailed to publicly-funded Australian maternal health dietetic services in May 2018. Quantitative and qualitative variables collected across preconception to postnatal services (including diabetes) included; births per year (BPY), number of beds, staffing (full time equivalents; FTE), referral processes, and models of care. Results were collated in > 5000; 3500 and 5000; and < 3500 BPY.

**Results:**

Forty-three eligible surveys were received from seven states/territories. Dietetic staffing levels ranged from 0 to 4.0 FTE (> 5000 BPY), 0–2.8 FTE (3500–5000 BPY), and 0–2.0 FTE (< 3500 BPY). The offering of preconception, antenatal and postnatal services varied significantly between hospitals (format, staffing, referral processes, delivery models). Few sites reported service effectiveness monitoring and only one delivered gestational diabetes mellitus care according to nutrition practice guidelines. Low staffing levels and extensive service gaps, including lack of processes to deliver and evaluate services, were evident with major concerns expressed about the lack of capacity to provide evidence-based care.

**Conclusions:**

Ten years after the initial survey and recommendations there remains an identified role for dietitians to advocate for better staffing and for development, implementation, and evaluation of service models to influence maternal nutrition.

## Background

Pre-conception and antenatal nutrition status and maternal dietary behaviours have long been recognised to impact maternal and child health outcomes, in both the short and long term [1, 2]. The Developmental Origins of Health and Disease (DoHAD) paradigm emphasises that environmental factors, including nutrition, interact with genotype variation, to affect the developmental trajectory accordingly [3]. In the pre-conception period, maternal weight and micronutrient status are associated with fertility, conception, and the child’s neurological status [4–6]. A poor-quality antenatal diet has been shown to increase the risk of inadequate or excessive gestational weight gain (GWG) [1, 2], preeclampsia [7], anaemia [8], preterm birth or miscarriage [9], gestational diabetes (GDM) and long term maternal overweight and obesity, type 2 diabetes mellitus and cardiovascular disease [10]. Moreover, it is also associated with negative child outcomes, including inadequate development [11], low birth weight [12], preterm birth [13], macrosomia [14], and an increased risk of chronic diseases later in life [10, 15]. Failure to lose pregnancy weight postpartum has been shown to impact on future pregnancies and to be a significant predictor of long-term maternal obesity up to 15 years later [16–19]. The impacts of diet on maternal and child outcomes highlights the importance of encouraging a high quality, evidence-based diet for all women in the pre-conception, antenatal and post-natal periods.

Dietitians have an essential role to play in providing effective medical nutrition interventions for prevention, as well as treatment of complications including GDM, diabetes in pregnancy, and suboptimal GWG [14, 20, 21]. Further, dietitians provide nutrition educational support to other health professionals. Traditionally dietitians in maternal health hospitals have worked primarily in the treatment of GDM and, more recently, in obesity management and for child-based services in neonatal intensive care and paediatric nutrition [22, 23]. To provide effective evidence-based care to optimise women’s and children’s health outcomes, services require operative dietetic service delivery models as well as evidence-based models of care. These should ideally incorporate, not only evidence based MNT, but systems that monitor the effectiveness of dietetic input.

The characteristics of women bearing children in Australia are changing with regards to sociodemographic variables such as age, family structure and cultural diversity and health related variables such as pre-pregnancy BMI and GDM prevalence [24]. Despite these changing demographics, the scientific evidence from the DoHAD literature and the publication of GDM and GWG guidelines [25], few effective maternal health dietetic service delivery models of care reported in the literature, beyond specific weight management programs [26, 27] and diabetes management guidelines [14, 22, 28–30]. There are calls for a greater attention to this “missed opportunity” in the provision of stronger public health and perinatal care approaches in Australia [31].

Prior to the expansion of the new Mater Mothers’ Hospital (MMH) in South East Queensland, Australia, in 2008, MMH dietitians conducted a cross-sectional survey of Australian maternal health dietetic services to inform the development of the MMH dietetic service [22]. Fifteen Australian maternal health dietetic services were surveyed (73% response), investigating staffing and service delivery and evidence-based models of care. Services reported low resourcing of dietetic maternal health care provision with considerable variation in staffing levels and service delivery. Individual antenatal inpatient and outpatient counselling dominated dietetic time and few used evidence-based models of care or guidelines. A role was identified for Australian maternal health dietitians to advocate for better staffing for nutrition related care and for implementation and evaluation of services with demonstrated health outcomes. This 2008 study advocated for service delivery improvements, including documented models of dietetic care.

Ten years on, it is timely to repeat this study to examine the provision of, and variations in, maternal health dietetic services particularly in light of the changing characteristics of childbearing women over this time. Hence, this current study aimed to examine dietetic service provision in Australian maternal health hospitals and identify models of dietetic service provision and care. This information will allow identification of service gaps and provide further data for the formation of service delivery improvements and models of care.

## Methods

A cross sectional survey methodology was employed targeting Australian publicly-funded maternal health dietetic services to quantify dietetic service provision and utilised models of dietetic service and care. The study is reported according to the STROBE statement guidelines for reporting observational studies [32]. This study received exemption from ethical approval from the Mater Research Institute – University of Queensland’s Human Research Ethics Committee (HREC/18/MHS/21). Participation was voluntary and consent assumed with survey return.

Hospitals were considered eligible for inclusion if they admitted women for the provision of antenatal services and were registered with the Women’s and Children’s Hospitals Australasia organisation. Hospitals external to Australia were excluded.

An online survey was informed by the prior survey [22] and sought information on the provision of services to maternal health patients from preconception to the postnatal period. The quantitative service variables included; number of births each year, number of beds, staffing levels (as full time equivalents, FTEs), referral processes, service delivery methods, models of care in use, service monitoring/quality processes used, and gestation stage when women were seen, and reason for referral. Each section provided opportunity for qualitative comment to allow a more detailed understanding of service provision.

Australian publicly-funded maternal health services were invited to complete the survey via an email link to an online survey portal (Survey Monkey) by the Women’s and Children’s Hospitals Australasia organisation (via maternal health services and allied health distribution lists). The survey link was also distributed via the statewide Maternity and Neonatal Clinical Network (Queensland) and the Agency for Clinical Innovation Nutrition in Hospitals Committee (New South Wales). These distribution channels provide a wide reach but do not allow numerical calculation of distribution. Recipients were encouraged to distribute the email to dietetic colleagues in maternal health services. Participation was voluntary and consent assumed with survey return. The survey was open for 6 weeks for completion with a reminder email posted at 1 month.

Quantitative responses were tabulated and descriptively summarised (mean, standard deviation, and median) in Microsoft Excel. When data were provided as a range the average was taken; responses of ‘minimal’ were classified as zero. Each hospital was provided a unique identifier for reporting purposes. Given the large variation in the tertiary hospital sizes and birthing numbers, results were collated in three groups of births per year (BPY): > 5000 births; between 3500 and 5000 births; and fewer than 3500 births. When more than one response was received from a hospital, the data were combined. When data were conflicting hospitals were contacted for clarification. Qualitative responses were coded and thematically analysed [33].

## Results

### Participating sites

Email invitations were distributed through the abovementioned channels in May 2018. The survey period ran for 6 weeks, with a reminder sent at 1 month. Fifty-six surveys were completed with 13 excluded from analysis, resulting in 43 responses with eligible data. Reasons for exclusion included not being an Australian service (*n* = 1; New Zealand); insufficient data on hospital births (*n* = 1), and multiple submissions from the same hospital (*n* = 11). Not all questions were answered resulting in missing data. A summary of the hospitals and resourcing are included in Table 1.

**Table 1:** Summary of dietetic staffing levels and service types in Australian maternal health hospitals

Category of births/year	Births/year (mean ± SD (median))	Hospitals (n)	State/Territory	Hospital location	Number beds (AN;PN) (n)	Maternal health dietetic FTE (n)
**> 5000**	6300 ± 1591 (6000)	11	1 New South Wales 3 Queensland 5 Victoria 2 Western Australia	11 metropolitan	Mean AN = 28.4 ± 16.5 (*n* = 9) Median = AN 30 Range = 3–50 N/A = 2 Mean PN = 42.3 ± 17.6 Median PN = 41.5 Range = 20–76 N/A = 1 AN/PN^a^ = 170 (*n* = 1)	Mean = 1.3 ± 1.6 Median = 0.2 Range = 0–4
**3500–5000**	4100 ± 572.8 (3950)	12	2 Australian Capital Territory 3 New South Wales 2 Queensland 2 Victoria 2 South Australia 1 Western Australia	10 metropolitan 1 regional 1 N/A	Mean AN = 30 ± 18.2 (*n* = 11) Median AN = 15.5 Range = 4–35 Mean PN = 38 ± 23.6 Median PN = 32 Range = 25–100 AN/PN^a^ = 30 (*n* = 1)	Mean = 1.1 ± 1.3 Median = 0.65 Range = 0–2.8
**< 3500**	1300 ± 1012.6 (1300)	20	6 New South Wales 6 Queensland 1 Victoria 1 South Australia 3 Tasmania 3 Western Australia	5 metropolitan 9 regional 6 rural	Mean AN = 13.8 ± 16.0 (*n* = 17) Median AN = 5 Mean PN = 16.4 ± 13.4 Median PN =15 AN/PN^a^: 13, 32 (*n* = 2) 5 country health beds (*n* = 1)	Mean = 0.6 ± 0.6 (*n* = 14) Median = 0.3 Range = 0–2 N/A (*n* = 2) Included in part of general funding (*n* = 4)

*AN* antenatal, *N/A* not available, *PN* postnatal

^a^ combined AN & PN service

The data from 43 hospitals spanned seven states and territories with 26% (*n* = 11) from hospitals with more than 5000 BPY, 28% (*n* = 12) 3500 to 5000 BPY and 46% (*n* = 20) less than 3500 BPY (Table 1). The majority of the metropolitan hospitals (*n* = 26) were from the larger two hospital groups with the rural and regional hospitals (*n* = 16) predominately from the smallest hospital category.

### Dietetics staffing levels

Dietetic staffing levels in maternal health services ranged from 0 to 4.0 FTE (median 0.2 FTE; mean 1.3 ± 1.6 FTE) in hospitals with > 5000 BPY, 0–2.8 FTE (median 0.65 FTE; mean 1.1 ± 1.3 FTE) between 3500 to 5000 BPY, and 0–2.0 FTE (median 0.3 FTE; mean 0.6 ± 0.6) with fewer than 3500 BPY (Table 1). In the hospitals with < 3500 births this FTE was generally reported as part of a larger and more general workload.

### Inpatient referral patterns

Similar patterns for inpatient referrals were noted across all hospital sizes (Additional file 1). In hospitals with > 5000 BPY referrals were received from midwifery (*n* = 4) or all health professionals (midwifery; doctor; blanket; diabetes service) (*n* = 3). Two services noted no dietetic funding, with obstetrics or midwifery contacting if deemed urgent. In services with 3500–5000 BPY, referrals were received from all health professionals (doctor, midwifery, dietetic assistant) (*n* = 1); blanket (*n* = 1); consultant only (*n* = 1); and midwifery only (*n* = 9). Those hospitals with < 3500 BPY, referrals were received from the antenatal team, via hospital systems (*n* = 2); consultant only (*n* = 2); or midwifery only (*n* = 10).

### Outpatient services

Maternal health related outpatient services, including antenatal, GDM and diabetes in pregnancy (DIP), were reported in 31 hospitals (72%) with 82% (*n* = 9) for hospitals with > 5000 BPY, 75% (*n* = 9) in hospitals with 3500–5000 BPY, and 65% (*n* = 13) in hospitals < 3500 BPY (Table 2). This also shows the FTE and number of women seen per clinical area, with antenatal provided as a summary (including GDM, DIP where separate data were unavailable) or by specific area. Wide variation existed in FTEs and number of women seen in each of the services, with higher levels of staffing and associated service activity in the hospitals with between 3500 and 5000 BPY. Many respondents noted that their current dietetic staffing levels were inadequate to meet the nutrition needs of women, especially for women with co-morbidities.

**Table 2:** Summary of work area FTEs and service activity across hospital sizes

Births/ year	Variables	Preconception	Antenatal outpatients (combines AN, GDM, DIP) – separate data unavailable	Antenatal	Gestational Diabetes Mellitus	Diabetes in Pregnancy	Postnatal
**> 5000**	**Respondents**	4	7	2	2	2	2
	**Dietitian FTE** (mean ± SD (median))	0.13 ± 0.04 (0.13)	0.45 ± 0.9 (0)	0.25, 1.5 (includes GDM)	0.6 (*n* = 1); in AN clinic FTE (*n* = 1)	0.3 ± 0.3 (0.3)	0.3 ± 0.3 (0.3) 170 ± 155.6 (170)
	**Women seen** (mean ± SD (median))	86.7 ± 70.9 (100)	630 ± 1081.2 (60)	1800, 1000 (includes GDM)	800 ± 0	317.5 ± 279 (317.5)	
**3500–5000**	**Respondents**	3	7	2	3	2	0
	**Dietitian FTE** (mean ± SD (median))	0.7 ± 0.6 (0.6)	0.48 ± 0.44 (0.35)	0.65 ± 0.9 (0.65)	1.1 ± 0 (1.1)	0.1 ± 0.1 (0.1)	
	**Women seen** (mean ± SD (median))	30 (*n* = 1) N/A (*n* = 2)	753 ± 287.9 (800)	520 (*n* = 1) N/A (*n* = 1)	520 ± 28.3 (520)	40–50 (*n* = 1) N/A (*n* = 1)	
**< 3500**	**Respondents**	3	10	3	6	3	0
	**Dietitian FTE** (mean ± SD (median))	0.75 ± 0 (*n* = 2); part of diabetes funding (*n* = 3)	0.2 ± 0 (*n* = 8); within general OPD (*n* = 2)	0	0.3 ± 0.2 (0.35)	0.03 (*n* = 1);in diabetes dietitian load (*n* = 1); in GDM load (0.1)(*n* = 1) < 12 (*n* = 1); N/A (*n* = 2)	
	**Women seen** (mean ± SD (median))	11.1 ± 2.0 (10)	140.8 ± 205.4 (50)	0	396.3 ± 177.5 (339)		

*AN* antenatal, *DIP* diabetes in pregnancy, *FTE* full time equivalents, *GDM* gestational diabetes mellitus, *N/A* not available, *OPD* outpatient department, *PN* postnatal

*“Unfortunately, we do not have a dedicated dietitian for our women. We are able to refer to a general ward dietitian when necessary but my experience is that this is very rarely used. It would be great to have a dedicated service.”* Participant (54) dietetic service >5,000 BPY

*“With close to 4000 births per year in (Australian city) there is an INSANE lack of dietetic support for women during pre, during and postnatal care. GDM is relatively well covered, however, women with other co-morbidities do not have access to sufficient dietetic support.”* Participant (15) dietetic service 3,500-5,000 BPY

It was noted that many women did not receive dietetic appointments despite their need with alternate services sometimes offered including community dietetic services, government telephone services or pamphlets.

*“We only see antenatal patients if referred to our general outpatient clinics. Cannot always see patients in a timely manner due to waiting times for outpatient appointments.”* Participant (52) Dietetic service <3,500 BPY

*“We do not see pregnant women with a high BMI, we refer to “Get Healthy in Pregnancy” (state- based telephone coaching service)”.* Participant (32) dietetic service <3,500 BPY

Models of funding were often not clear to the dietetic services themselves with maternal health care often absorbed as part of general funding inpatient and outpatient models.

The offering of preconception, antenatal services and post-natal dietetic services varied significantly between hospitals (Table 2). Preconception services were offered by 10 (23%) of hospitals, equally spread across hospital size. Preconception services in hospitals with > 5000 BPY were delivered as 1:1 appointments (*n* = 3), as 1:1 and groups (*n* = 3) in hospitals with 3500–5000 BPY, and as 1:1 (*n* = 2) and 1:1 and groups (*n* = 2) in hospitals < 3500 BPY. Hospitals offered 1:1 antenatal appointments (in person or telehealth (*n* = 1)) and/ or group education.

### GDM services

As shown in Additional file 1, only one site (> 5000 BPY) reported delivering GDM care according to the Journal of the Academy of Nutrition and Dietetics Nutrition Practice Guidelines [34–36]. These are defined as one new individual appointment in the week following referral and two or more review appointments with the first review within a week of the first appointment. One hospital with 3500–5000 BPY and two with < 3500 BPY offered the correct number of appointments but not in the appropriate format (e.g. group setting) and/or not in the correct time frame specified. In general, GDM appointments were offered as individual appointments and/ or groups for both initial and review appointments. No other guidelines were reported being followed.

*“We see GDM women as a group session only – they are hard to give an estimate (of time provided) as it is not really a dietetic consult.”* Participant (30) dietetic service > 5,000 BPY

GDM dietetic services were auspiced under different services in different hospital from antenatal, general medicine, community and diabetes services.

*“GDM & DIP service is separate stream from antenatal and community”* Participant (25) dietetic service < 5,000 BPY

Many appointments schedules were not offered within the times stipulated in the Academy of Nutrition and Dietetics Nutrition Practice Guidelines [36]. One site did not offer reviews unless women commenced insulin. Times for initial appointments ranged from 30 to 120 min and reviews ranged from 15 to 60 min.

### Diabetes in pregnancy services

The majority of DIP appointments at each site were individual (face to face, telehealth). The length of new appointments ranged from 30 to 60 min with review appointments ranging 30–60 min. Appointment numbers ranges from one to five throughout the pregnancy with the initial appointment time ranging from pre-pregnancy to 15 weeks.

### Postnatal services

Specific data on postnatal services were only provided by two sites (both > 5000 BPY). They offered individual appointments (*n* = 1) or individual or telehealth services (*n* = 1) for 30 min new and review appointments. A number of respondents expressed the view that the demand for postnatal services was low or may be better placed external to the acute hospital setting.

*“No routine postnatal appointments (are offered) as low demand.”* Participant (38) dietetic service >5, 000 beds

*“(I am) not convinced an acute care facility is the right place for postnatal follow up for the majority of deliveries. Think we need to be more innovative and accommodating to the needs of women post-delivery.”* Participant (26) dietetic service 3,500-5,000 BPY

As noted only two respondents provided data about postnatal services (Table 2 and Additional file 1). However, only one hospital had the capacity to offer services to these women through a dietitian-led clinic which received referrals from the antenatal clinic. These women were those who had GDM, entered pregnancy with a BMI > 25 kg/m^2^ or experienced excessive GWG and were seen predominantly through a telehealth model of care. The other respondent managed these women through a university dietetic-clinic partnership due to low demand and decreased hospital accessibility and time pressures.

### Outpatient referral patterns

Models for referral to maternal health dietetic services ranged from self-referral to blanked referral with most services accepting referrals from medical and midwifery staff. All centres accepted GDM and DIP outpatient referrals through various care pathways and models of care involving diabetes educators, endocrinologists, obstetric medicine physicians and obstetricians. This was similar for DIP.

### Adherence to clinical practice guidelines

Only one site (> 5000 BPY) identified following of best practice clinical guidelines [36]. The lack of Australian nutrition clinical practice across maternal health care was highlighted in the qualitative data.

*“There are no Australian clinical practice guidelines that cover the conditions seen in our maternity diet clinics therefore (it is difficult to) estimate resources.”* Participant (38) dietetic service 5000 BPY

### Outcome monitoring for service effectiveness

The clinical outcomes for monitoring service effectiveness included changes in body mass, diet quality and medication use. Process measures included attendance rates and composite scores of best practice.

## Discussion

This survey of dietetic service provision in maternal health hospitals has demonstrated the extremely wide variation in service delivery models and service capacity across Australia. Critically low levels of resourcing and numerous service gaps have been identified, including minimal evidence-informed processes in place to routinely monitor and evaluate service delivery. The qualitative methodology emphasised the significant concern that dietetic services have regarding the inability to be able to provide evidence-based nutrition care to women.

The experience of this, and the prior study, shows that few maternal health hospitals have been able to address the inadequate levels of dietetic service provision or have undertaken systematic development and delivery of maternal health dietetic services over the past 10 years [22]. Over the 10 years there has been an approximate increase of one FTE in the two larger hospital groupings and only by 0.5 FTE across the smaller sites. While there were far more respondents in the second survey there remained a lack of defined models of care or staffing to meet the evolving needs of services reflecting the changed needs of women of childbearing age. Reasons for this may include nutrition lacking priority with maternal health hospital management, the lack of capacity of dietitians to enact systemic service changes or advocate for changes in under-resourced and under-prioritised areas, and/or a lack of locally relevant guidelines that could inform the resourcing and practice of maternal health dietitians.

Whilst preconception and postnatal services may not be priority areas for hospital based maternal health dietitians, all areas of antenatal nutrition with demonstrated health outcomes should be within their scope of practice and service delivery. For example, it is well recognised that MNT is a cornerstone treatment in GDM [36]. Despite the lack of Australian GDM guidelines, American nutrition practice guidelines recommend women with GDM receive MNT according to an evidence-based dietetic appointment schedule, with a minimum of a one-hour individual initial session and two review appointments. This has demonstrated reduction in medication requirements and better blood glucose control [34].

Calculating staffing levels from these guidelines provides direction for planning and allows comparison with current services. For example, a hospital with 5000 BPY with an average 10% GDM diagnosis [37] equates to 500 women with GDM annually. To ensure guideline provision of a minimum of an individual initial session (60–90 min) and two review appointments (2 × 30 min), 0.63–0.76 FTE is required. This staffing level is for direct care only and does not account for case preparation, write up and conference. Given a dietetic mean of 1.3 ± 1.6 EFT reported in this study for hospitals this size, dietetic departments require significantly more funding to meet just this one antenatal nutrition guideline.

The lack of Australian based nutrition antenatal clinical guidelines was evident in both the quantitative and qualitative data reporting. Australian nutrition practice guidelines for maternal health, developed or endorsed, are urgently required and have been called for by others [31]. The science behind the epigenetics implications and multi-generational negative health outcomes of poor nutrition in the preconception, antenatal and post-natal periods is well established [38]. Best practice, protocol driven recommendations for the management of nutrition across the antenatal to related spectrum will extend this science to enable implementation of models of care workforce models including alternate models such a digital health [39], and routine monitoring and evaluation of outcomes and services.

A strength of this study is the continued, longitudinal mapping of the Australian nutrition-related maternal health services. Additionally, the large response rate and the spread of respondents across states and territories (except for the NT) and metropolitan, regional and rural services provide weight to the responses obtained. Compared with the previous survey [22] this received almost five times the responses which may reflect a greater number of services providing maternal health services and the distribution method of email rather than targeted mail. However, this also potentially introduces a self-selection bias. Online invitation through a distribution mailing list may also be considered a limitation, preventing the calculation of response rate. The addition of qualitative comments in this iteration has provided additional insights into the challenges faced by services in providing evidence-based care.

Targeting maternal health services may also have reduced the potential services delivering diabetes-related care through endocrinology or general medicine services who may not have received the emailed invitation. A further limitation relates to the inability to provide ‘meaningful’ service planning data. The calculation of FTE/100 births was prevented due to the qualitative nature of some service capacity data provided by respondents.

## Conclusions

This paper described publicly-funded Australian maternal health dietetic service models and capacity. Low levels of resourcing and considerable staffing levels and service variation still exists, 10 years on from the initial survey. As highlighted in our previous paper, across all services there is exists an identified role for maternal health dietitians to advocate for better staffing and for the urgent development, implementation and evaluation of services (and models of care) to influence preconception, antenatal and postnatal nutrition.

## Supplementary information

**Additional file 1.** Summary of processes, and monitoring, and adherence to best practice guidelines across different hospital sizes.

## Data Availability

The datasets used and/or analysed during the current study are available from the corresponding author on reasonable request.

## Abbreviations

AN: Antenatal
BMI: Body mass index
BPY: Births per year
DIP: Diabetes in pregnancy
DoHAD: Developmental origins of health and disease
FTE: Full time equivalents
GDM: Gestational diabetes mellitus
GWG: Gestational weight gain
MNT: Medical nutrition therapy
N/A: Not applicable
NT: Northern Territory
OPD: Outpatient department
PN: Postnatal
