# Supplementary material for: Nutrition and maternal health: a mapping of Australian dietetic services
Source: BMC Health Serv Res. 2020 Jul 16;20:660. doi: 10.1186/s12913-020-05528-4 (PMC7364531; doi:10.1186/s12913-020-05528-4)
Supplement: Supplementary file 1 — Additional file 1. Summary of processes, and monitoring, and adherence to best practice guidelines across different hospital sizes. [file 12913_2020_5528_MOESM1_ESM.docx]

**Additional file 1.** Summary of processes, and monitoring, and adherence to best practice guidelines across different hospital sizes.

| **Births/ year** | **Variables** | **Preconception** | **Antenatal** | **Gestational Diabetes Mellitus** | **Diabetes in Pregnancy** | **Postnatal** |
| --- | --- | --- | --- | --- | --- | --- |
| **>5000** | **Respondents (n)**  **Adherence to best practice guidelines**  **Clinic processes**  **Postnatal linkage**  **Clinical outcomes for monitoring**  **service effectiveness**  **Process outcomes for monitoring service effectiveness** | 4  N/A  Integrated into MD clinic (n=1)  Referrals from endocrinologists (n=1); fertility and endocrinologists (n=1)  N/A  Weight change and diet quality change (n=1)  OOS; attendance and cancellation rates (n=1) | 2  N/A  MD/ANC (n=2)  Referrals via clinic triage form (n=1); prompts in maternity record (n=1)  Made by ANC dietitian at K36 (n=1); Nil; (n=1); if requested (n=1)  Overall GWG; diet quality; fruit and vegetable intake (n=1)  OOS; attendance and cancellation rates (n=1) | 2  1 (at one site)  MD clinic (n=2)    Referred by DE and obstetric medicine (n=4)  Made by ANC dietitian at K36 (n=1); Nil (n=1)    Diet versus medication controlled (n=1)  Appointments delivering best practice (i.e. 1 new; 2+ reviews) (n=1) | 2  N/A  MD clinic (n=2)    Referred by DE,  endocrinologist or obstetrician (n=3)    As required (n=1); Nil (n=1)    Nil  K of visit; OOS; attendance and cancellation rates (n=1) | 2  N/A  Dietetic clinic (n=1)  Referral by ANC and GDM dietitians (n=1)  Nil  Weight change; diet quality change (n=1)  OOS; attendance and cancellation rates (n=1) |
| **3500- 5000** | **Respondents (n)**  **Adherence to best practice guidelines**  **Clinic processes**  **Postnatal linkage**  **Clinical outcomes for monitoring**  **service effectiveness**  **Process outcomes for monitoring service effectiveness** | 3  N/A    Dietitian-only clinic (n=1); MD clinic (n=1)  Self-referral (n=2)  Pregnancy; weight loss (n=1)  Nil | 2  N/A  Nil (n=1)  Outpatient partner services (n=1)  Clinical outcomes (n=1)  Reach, uptake, satisfaction (n=1) | 3  Guideline number of consults (n=1)  MD clinic (n=2)  Blanket referral (n=1); DE and dietitian in group (n=1); GP, midwife, doctor (n=1); DE, midwives, endocrinologists (n=1)  Nil (n=1); Outpatient partner services (n=1)  Commencing pharmacotherapy (n=1)    Guideline number of appointments and timing (n=1); referrals seen in 10 working days (n=1) | 2  N/A  MD clinic (n=2); maternity (n=1); endocrinology service (n=1)    Intake midwife and/or dietitian (n=2); DE, endocrinologists, midwives (n=1)    Nil (n=1); T1DM only (n=1)  Nil (n=1) | 0  0 |
| **<3500** | **Respondents (n)**  **Adherence to best practice guidelines**  **Clinic processes**  **Postnatal linkage**  **Clinical outcomes for monitoring**  **service effectiveness**  **Process outcomes for monitoring service effectiveness** | 3  N/A  MD clinic (n=2); dietitian-only clinic(n=2)  Blanket referral (n=2); preconception screening tool (n=1)  Nil  HbA1c; diet quality (n=1)  Nil | 3  N/A  General clinic (n=1)  Nil  Nil  Nil | 6  Guideline number and timing of appointments (n=5)  Endocrinology clinic (n=4); maternity clinic (n=1)  Blanket referral (n=1); DE (n=2); clinic nurse, GP obstetricians or endocrinologist (n=4)  Nil  Neonatal outcomes; insulin requirements (n=11)  Nil | 3  N/A  Endocrinology clinic (n=2); maternity clinic (n=1)    Nil  GDM BGL targets (n=1); neonatal outcomes, insulin requirements, maternal weight gain (n=1)  Nil | 0 |

ANC, antenatal clinic; BGL, blood glucose levels; DE, diabetes educator; GP, general practitioner; GWG, gestational weight gain; K, weeks of pregnancy; MD, multidisciplinary; OOS, occasions of service; PN, postnatal; T1DM, type 1 diabetes mellitus; T2DM, type 2 diabetes mellitus
